# Prevalence and Determinants of Anemia among Children in Zanzibar, Tanzania: Analysis of Cross-Sectional Population Representative Surveys

**DOI:** 10.3390/children8121091

**Published:** 2021-11-25

**Authors:** Fatma Ally Said, Ahmed Gharib Khamis, Amanat Habib, Hexiang Yang, Zhangya He, Xiaoqin Luo

**Affiliations:** 1 Department of Nutrition and Food Safety, School of Public Health, Xi’an Jiaotong University, Xi’an 710061, China; datuu67@yahoo.com (F.A.S.); amanathabib@yahoo.com (A.H.); clyeah@hotmail.com (H.Y.); hzy1129185047@stu.xjtu.edu.cn (Z.H.); 2 Department of Preventive Services and Health Promotion, Ministry of Health Zanzibar, Zanzibar, Tanzania; 3 Department of Epidemiology and Biostatics, Muhimbili University of Health and Allied Science, Dar-es-salaam, Tanzania; ahmadboycd@gmail.com

**Keywords:** anemia, prevalence, determinants, children, Zanzibar, Tanzania

## Abstract

The aim of this study was to assess the prevalence of anemia and its determinants among children aged 6–59 months in Zanzibar, Tanzania, from 2005 to 2015. We used secondary data from the Tanzania Demographic and Health Surveys from three different periods: 2005, 2010, and 2015. A sample of 3502 child-mother pairs from three survey datasets was used to analyze the overall prevalence of anemia and reveal its determinants. Hierarchical logistic regression was used to demonstrate the prevalence odds ratio of factors, both from the mothers and children, for anemia prevalence. The study indicated a significant decrease in anemia prevalence from 76.1% in 2005 to 65.4% in 2015 (*p* < 0.001). Hierarchical logistic regression between variables and anemia showed a significant association (*p* = 0.02) with households that improperly disposed of stool, children with minimum dietary diversity (*p* = 0.041), children in low age quartiles (*p* = < 0.001), and underweight children (*p* = 0.025). Maternal, household characteristics, and child factors were associated with childhood anemia in Zanzibar, Tanzania. Despite the significant decrease of anemia in Zanzibar, the overall prevalence rate is still a significant public health concern. Designing and strengthening comprehensive interventions to address anemia in the general population and different categories should be given special consideration.

## 1. Introduction

Anemia is a global public health problem affecting one-third of the world’s population both in developed and developing countries, with significant consequences for human health as well as for social and economic development [1,2]. Anemia is a condition defined as a considerable reduction of hemoglobin concentration or reduction of circulating red blood cells below the normal range, making it insufficient to meet an individual’s physiological needs [3,4,5].

Though anemia affects people of all ages, pregnant women and children are more affected. It was estimated that 42% of children less than five years of age and 40% of pregnant women are anemic globally. It is a significant public health problem associated with increased morbidity and mortality in women and children [6,7,8], poor birth outcomes [9], decreased work productivity in adults [10], and impaired cognitive and behavioral development in children [11]. The most significant contributor to the onset of anemia is iron deficiency due to insufficient iron intake through diet, iron mal-absorption or elevated demand for iron due to abrupt growth [12,13]. It was reported that 50% of the cases of anemia have iron deficiencies but more often in coexistence with a variety of other causes that can be isolated [14].

According to the anemia regional disparities report, one-third of the Tanzanian population is anemic, and Zanzibar reported a slight reduction in childhood anemia prevalence of nearly 10% [15]. However, the overall situation needs to be observed as Tanzania Demographic and Health Surveys concluded that 65% of children under 5 were anemic in Zanzibar [16]. Over the years, Tanzania has been struggling and has made tremendous economic transformations that have decreased the underlying form of malnutrition. However, Zanzibar, a autonomous country in the United Republic of Tanzania, is still suffering from a high prevalence of anemia in children, 15 points more than that of mainland Tanzania [15]. In addition, information on anemia determining factors other than those related to iron deficiency is minimal. The current study was carried out to establish information on the trend of anemia prevalence and mainly its determinants in children between 6–59 months in Zanzibar to be used by the policymakers and nutrition practitioners.

## 2. Materials and Methods

### 2.1. Study Design

The secondary data analysis utilized three Tanzania Demographic and Health Surveys (TDHS) conducted in 2005, 2010, and 2015, available from the online portal of demographic health programs (www.dhsprogram.com, access date: 16 January 2020). These national representative surveys employ cross-sectional study designs to measure levels, patterns and trends in demographic and health indicators and are conducted every five years in all regions of mainland Tanzania and Zanzibar, by the National Bureau of Statistics (NBS) in mainland Tanzania and the Office of the Chief Government Statistician (OCGS) in Zanzibar.

We analyzed three datasets, TDHS 2005, 2010, and 2015, to determine the prevalence of anemia in children because they have the variables needed for the analysis, and the data was collected in a similar manner [16]. The three datasets were only used for analysis to determine the prevalence and only the TDHS dataset of 2015 was used to determine the correlation of anemia in children, aiming to avoid missing variables across three data sets. The three data sets used for analysis had a total sample of 3502 child-mother pairs and of them 1147 were from 2005, 1182 were from 2010, and 1173 were from 2015.

### 2.2. Dependent Variable

The primary outcome of interest was anemia. In all three TDHS surveys, anemia was measured as hemoglobin (Hb) concentration in the blood. Blood samples were drawn from a drop of blood taken from a finger prick and collected in a micro cuvette. Hb level analysis was carried out on-site using a battery-operated portable Hemo-Cueanalyzer, and results were obtained on the spot. The cutoff points of anemia in TDHS were less than 110 g/L according to the cut off points for anemia recommended by WHO.

### 2.3. Independent Variables

Independent variables included child age in months, child’s sex, birth weight in grams, feeding practices of the children, diarrhea, fever, vitamin A supplementation, iron supplementation, deworming, and anthropometric indices (stunting, wasting, and underweight). Other independent variables were the caregiver’s educational level, maternal anemia, number of children living in the house, maternal age, residence and wealth index, sex, and age of the head of the household. The anthropometric measurements were taken using a standard weighing scale and height board.

### 2.4. Data Analysis

Analysis of data was done using SPSS (IBM Version 23.0, SPSS Inc., Chicago, IL, USA). Descriptive statistics were used to describe the characteristics of the study population. We used Pearson Chi-square tests to compare the prevalence of anemia between three years and between ages categories, where we used age quartiles to decrease the bias.

A hierarchical regression model was used to determine factors associated with anemia. The association was measured using the recent survey dataset of 2015, aiming to avoid missing variables across the three surveys. Variables were categorized into three models: household factor as the first model, with a *p*-value > 0.005, maternal factors as the second model, which had a *p*-value > 0.005, and child factors as the third model.

Association between dependent and independent variables was determined using odds ratios (OR) at *p* < 0.05 with a 95% confidence interval.

## 3. Results

### 3.1. Descriptive Characteristics of the Children, 2005–2015

Results revealed that more than 70% of the interviewed households were from rural areas in all three surveys. The proportion of interviewed households in rural areas was 82.2%, 75.2%, and 81.0% in 2005, 2010, and 2015, respectively. The respondents’ wealth index showed that 35.6%, 32.0%, and 38.4% of the respondents in 2005, 2010, and 2015, respectively, were richer. Each survey showed that the richer group of respondents were older than any other group. There was an increase in maternal educational attainment at the level of secondary education, which was 37.1% in 2005 and improved to 50.0% in 2015. More than 90% of the mother respondents were married, and more than 80% of the households were male-headed. In all three datasets, most of the interviewed households had between 4 and 10 people in their households. Meanwhile, anemia decreased with the increasing wealth index of the respondents (*p* for tend = 0.004) and anemia also decreased with an increasing BMI of the mother respondents (*p* for tend = 0.01).(Table 1).

### 3.2. The Trend in the Prevalence of Anemia among 6–59-Month-Old Children in Zanzibar

As shown in Table 2, the prevalence of anemia decreased significantly over 15 years, from 76.1% in 2005 to 65.4% in 2015 (*p*-value < 0.001), almost 10%.

### 3.3. Prevalence of Childhood Anemia Adjusted between Ages

The prevalence rate was high in the low age quartile across all surveys. Among 1147 individuals interviewed in 2005, the prevalence of anemia was high, 29.2% in the Q1 age group, and the least was 21.4% in the Q4 age group. In 2010, 30.9% of the anemic children were in the Q1 age group, while 20.1% were in the Q4 age group. In 2015, 33.4% of the anemic children were in the Q1 age group and the least, 18.1%, in the Q4 age group. The rate of anemia was revealed to be high in the Q1 age group across all surveys (Table 3).

### 3.4. The Association of Household Characteristics and Maternal Characteristics with Anemia among Children

Table 4 shows the results of hierarchical logistic regression analysis of anemia. The results reveal that households that improperly disposed of stool were at risk of getting anemia (aβ = 1.44, 95% CI: 1.06–1.94, *p* = 0.02), compared to households with a proper stool disposal system. No other household and maternal factors were revealed to have an association with childhood anemia.

### 3.5. The Association of Child Characteristics with Anemia among Children

We finally analyzed the association of children’s characteristics with anemia (Table 5). It was revealed that children in the lower age quartile were found to be more likely to have anemia (aβ = 0.18, 95% CI: 0.12–0.28, *p* < 0.001) and (aβ = 0.30, 95% CI: 0.20–0.44, *p* < 0.001), and those children with minimum dietary diversity (MDD) were more likely to have anemia (aβ = 0.61, CI = 0.37–0.98, *p* = 0.045) compared to their counterparts. Underweight children were also at higher risk of having anemia (aβ = 1.54, 95% CI: 0.94–2.52, *p* = 0.025).

## 4. Discussion

Childhood anemia is still a public health threat in Zanzibar. The findings showed that the overall prevalence of anemia is 65.4%, and the prevalence of anemia decreased significantly over 15 years, from 76.1% in 2005 to 65.4%. The prevalence rate was high in the low age quartile. There is a positive association between anemia and households with improperly disposed stool, children with minimum dietary diversity, children in low age quartiles, and underweight children.

The reported prevalence is similar to the national reported prevalence of 65% [16]. This prevalence was found to decrease when compared with the prevalence of 2005, which was 74.1%. The higher prevalence was very similar to the prevalence reported in the three districts of the Arusha region with 84.6% [17] and the Mwanza region with 77.2% [18]. It has been reported that children are fed with staple foods rich in carbohydrates like rice, maize, and cassava as the main meal and fail to incorperate iron-rich food in their meal [15]. In accordance with a previous study [19], our results also indicated that inappropriate feeding practices, which lead to nutritional inadequacy, influence the reported anemic prevalence in Zanzibar. The reported prevalence was also influenced by poverty, as it was highlighted that most forms of undernutrition continue to persist in Zanzibar, with children from impoverished households being three times more at risk. Again, there were some gaps in the provision of nutrition services. For example, less than 10% of women took iron supplements during pregnancy [20], contributing to child anemia after delivery. This was consistent with the reported high prevalence of maternal anemia in Zanzibar of 43.2% [21].

The burden of child anemia has significantly declined for the past fifteen years from 74.1 to 65.0%. It was undoubtedly due to improved health care services and increased deworming coverage to over 90% in under-five children across all Zanzibar [22]. However, the malaria prevalence rate decreasing to less than 1% [16] contributed greatly to this notable reduction of anemia prevalence in children. Despite this significant decline, the anemia prevalence was still high and unacceptable, which may affect the intellectual capacity of children.

The analysis also revealed the disparities of prevalence rates in different age quartiles. The prevalence among young children was higher than that of all other age groups across all years. This situation was likely due to inappropriate feeding practices that lead to an insufficient daily intake of iron by children [23], as it was reported that in Zanzibar, only 18% of children receive diversified food and those with a minimum acceptable diet is reported to be less than 30% [21]. Early initiation of complementary food, inadequate breastfeeding, and susceptibility to infection affect essential micro-nutrients bioavailability from the diet consumed by Zanzibar children [19]. The findings were very similar to a survey done in Ethiopia, Ghana, and Bangladesh [24,25,26]. 

Our study revealed the association between anemia and households which dispose of stool improperly, children in the low age quartile, those with MDD, and those underweight. The likelihood of developing anemia was significantly associated with households that improperly disposed of stool, and this might be due to the exposure of helminth infections from stool that can contribute to the likelihood of anemia occurrence in children [27]. It has been claimed that most households have improper sewage systems, and there is no centralized sewage system in Zanzibar [28]. Households reported to have no toilet is 16%, with more than 30% reported to have inadequate toilet facilities in Zanzibar [29].

The children’s age was significantly associated with anemia; younger children were more at risk than the older ones. The results showed that those in low-age quartiles had increased risk. The observed association may be due to poor feeding practices, early weaning, inadequate breastfeeding, and susceptibility to infection. A report that was written by Kinabo et al., in 2012, showed that there were problems of early weaning in children in Zanzibar, as the mean age of the weaning period was 4 months. There was also a lack of knowledge on the best complementary feeding practices in mothers [19]. The report insisted that the problem was due to a lack of awareness and limited information on child feeding. It was also about the poor food preparation and feeding skills, thus solidifying why younger children are at more risk of developing anemia [21].

The results showed a strong correlation between anemia and MDD, while MDD was associated with anemia in children due to inadequate food diversification. Although Zanzibar has a wide variety of local foods suitable for feeding children, in this study, only 18% had a MDD. In addition, mothers/caregivers lacked knowledge of how best to combine these foods to prepare energy- and nutrient-dense diets [19,21]. A number of reports have documented inappropriate skills of mothers in preparing complementary food and feeding practices as well [16,21,28].

Among the analyzed variables related to children, low weight for age (underweight) remained associated positively with anemia. Many other factors contributed to the association of anthropometric indices and anemia, including poverty, water, and sanitation. As reported, there is a flawed centralized sewage system in Zanzibar and no sanitary policies and caring practices with food insecurity [20]. This report was highlighted in Zanzibar’s nutrition policy and insisted that women did most agricultural activities with limited potential for crop and livestock production. In addition, there is negligible diversification of income-generating activities as most of the Zanzibar area is prone to environmental degradation, and their households are highly vulnerable to acute, chronic, and transitory food insecurity and malnutrition [28]. This observed association was also reported in other studies [30,31,32].

This study clarifies the prevalence of childhood anemia in Zanzibar, Tanzania, over 15 years, with some limitations to consider. The analysis of prevalence and trends included three surveys, and correlations were identified only from the most recent surveys and so older surveys may show different correlations compared to the most recent ones. However, the recent survey was used to avoid missing information on some variables that determine this association. Despite the above limitation, this study will provide insight into trends regarding anemia and will hopefully provide a clue on possible critical interventions for children in Zanzibar.

## 5. Conclusions

Anemia in children aged 6–59 months decreased across the years, but this improvement was still not convincing, and the higher rate was found in lower age categories. Lower age, underweight, given MDD, and improper stool disposal were associated with childhood anemia in Zanzibar. Special interventions, such as improving the addition of iron-rich foods, vitamin C supplementation to children, supporting deworming programs, and community promotion on good feeding practices with food diversification should be given serious consideration to address this problem.

## Figures and Tables

**Table 1:** Descriptive characteristics of the respondents, 2005–2015.

Variables	2005		2010		2015	
	Anemia N (%)	No Anemia N (%)	Anemia N (%)	No Anema N (%)	Anemia N (%)	No Anemia N (%)
**Residence of the respondent**						
Urban	153 (75)	51 (25)	198 (67.6)	95 (32.4)	141 (63.6)	80 (36.2)
Rural	719 (76.2)	224 (23.8)	632 (71.1)	257 (28.9)	638 (67)	314 (33.0)
**Wealth index of the respondents (*p* for tend = 0.004)**						
Poorest	85 (83.3)	17 (16.7)	52 (74.3)	18 (25.7)	11 (84.6)	2 (15.4)
Poorer	92 (74.8)	31 (25.2)	127 (71.8)	50 (28.2)	88 (73.3)	32 (26.7)
Middle	149 (79.3)	39 (20.7)	146 (71.2)	59 (28.8)	148 (70.1)	63 (29.9)
Richer	307 (75.2)	101 (24.8)	276 (72.4)	105 (27.6)	311 (69.0)	140 (31.0)
Richest	239 (73.3)	87 (26.7)	229 (65.6)	120 (34.4)	221 (58.5)	157 (41.5)
**The educational level of the mother respondents**						
No education	298 (76.8)	90 (23.2)	218 (71.7)	86 (28.3)	198 (72.3)	75 (27.7)
Primary	314 (75.8)	100 (24.2)	300 (69.4)	132 (30.6)	198 (67.1)	97 (32.9)
Secondary	240 (76.2)	75 (23.8)	308 (70.2)	131 (29.8)	376 (63.9)	212 (36.1)
Higher	20 (66.7)	10 (33.3)	4 (57.1)	3 (42.9)	9 (47.4)	10 (52.6)
**Marital status of the respondent**						
Single	7 (87.5)	1 (12.5)	9 (90.0)	1 (10)	10 (100)	0 (0)
Married/with partner	807 (75.8)	257 (24.2)	768 (69.8)	332 (30.2)	707 (65.8)	368 (34.2)
Separated/Divorced	58 (77.3)	17 (22.7)	53 (73.6)	19 (26.4)	62 (70.5)	26 (29.5)
**Sex of Head of household**						
Male	767 (76.3)	238 (23.7)	726 (70.8)	300 (29.2)	683 (67.3)	332 (32.7)
Female	105 (73.9)	37 (26.1)	104 (66.7)	52 (33.3)	96 (60.8)	62 (39.2)
**BMI of the mother respondent (kg/m^2^) (*p* for tend = 0.01)**						
Underweight	133 (77.3)	39 (22.7)	94 (75.2)	31 (24.8)	68 (72.3)	26 (27.7)
Normal	557 (77.3)	164 (22.7)	474 (72.4)	181 (27.6)	406 (69.8)	176 (30.2)
Overweight	120 (74.5)	41 (25.5)	184 (66.7)	92 (33.3)	184 (63.4)	106 (36.6)
Obesity	62 (66.7)	31 (33.3)	78 (61.9)	48 (38.1)	121 (58.5)	86 (41.5)
**Number of people living in the house**						
3 and below	72 (76.6)	22 (23.4)	59 (72.0)	23 (28.0)	59 (72.0)	23 (28.0)
4 to 10	746 (76.7)	226 (23.3)	702 (70.3)	296 (29.7)	645 (64.8)	350 (35.2)
above 10	54 (66.7)	27 (33.3)	69 (67.6)	33 (32.4)	75 (78.1)	21 (21.9)

**Table 2:** Trends in prevalence of anemia among 6–59-month-old children Zanzibar (2005–2015).

Year	Anemia	*p*-Value for Trend
2005	76.1	0.001
2010	70.2	
2015	65.4	

**Table 3:** Prevalence of childhood anemia adjusted between ages in Zanzibar 2005–2015.

Age Quartiles	2005		2010		2015	
	*n* = 1147 Anemia (*n* = 872)		*n* = 1182 Anemia (*n* = 830)		*n* = 1173 Anemia (*n* = 779)	
	*n* (%)	*p*-Value	*n* (%)	*p*-Value	*n* (%)	*p*-Value
Q1	255 (29.2)	<0.001	257 (30.9)	<0.001	260 (33.4)	<0.001
Q2	234 (26.8)		227 (27.3)		203 (26.1)	
Q3	196 (22.5)		176 (21.5)		175 (22.5)	
Q4	187 (21.4)		167 (20.1)		141 (18.1)	

**Table 4:** Hierarchical regression analysis of the association of household characteristics and maternal characteristics with anemia among children aged 6 to 59 months in Zanzibar, Tanzania.

Variables		Crude Odds Ratio (COR)		Adjusted Odds Ratio (AOR)	
		COR (95% CI)	*p*-Value	AOR (95% CI)	*p*-Value
**Household Characteristics**					
Residence	Urban	Ref		Ref	
	Rural	1.15 (0.85–1.56)	0.362	0.80 (0.56–1.13)	0.216
Gender of HHH	Male	Ref		Ref	
	Female	0.75 (0.53–1.06)	0.107	0.78 (0.55–1.12)	0.187
Age of the HHH (years)	18–29	Ref		Ref	
	30–49	0.72 (0.47–1.1)	0.131	0.82 (0.53–1.26)	0.373
	≥50	0.73 (0.47–1.16)	0.186	0.92 (0.57–1.47)	0.741
Household wealth	Middle	Ref		Ref	
	Poorest	2.34 (0.51–1.87)	0.277	2.64 (0.53–1.29)	0.233
	Poorer	1.17 (0.71–1.93)	0.537	1.2 (0.70–2.10)	0.503
	Richer	0.94 (0.66–1.35)	0.758	1.03 (0.69–1.54)	0.863
	Richest	0.59 (0.41–0.86)	0.005	0.76 (0.47–1.21)	0.252
Presence of iodized salt	Yes	Ref		Ref	
	No	0.69 (0.53–0.91)	0.008	1.43 (0.55–3.71)	0.455
Stool disposal	Properly disposed	Ref		Ref	
	Improperly disposed	1.53 (1.15–2.05)	0.004	1.44 (1.06–1.94)	0.020
Water sources	Protected	Ref		Ref	
	Unprotected	1.18 (0.53–2.62)	0.674	1.22 (0.54–2.75)	0.632
**Mother Characteristics**					
Mother’s BMI	Underweight	Ref		Ref	
	Normal	0.88 (0.54–1.43)	0.612	0.97 (0.58–1.64)	0.938
	Overweight	0.66 (0.39–1.11)	0.116	0.88 (0.51–1.52)	0.646
	Obesity	0.54 (0.32–0.91)	0.022	0.79 (0.45–1.41)	0.431
Age of mother (years)	<18	Ref		Ref	
	18–35	0.60 (0.43–0.84)	0.003	0.83 (0.57–1.21)	0.332
	>35	0.53 (0.35–0.82)	0.004	0.87 (0.53–1.45)	0.608
Mother’s occupation	Not working	Ref		Ref	
	Self-employment	0.93 (0.59–1.46)	0.764	0.94 (0.58–1.53)	0.832
	Farmers	1.08 (0.66–1.77)	0.762	1.27 (0.74–2.17)	0.384
	Employed	0.66 (0.51–0.87)	0.003	0.81 (0.61–1.09)	0.174
Education of mother	No education	Ref		Ref	
	Primary	0.78 (0.54–1.12)	0.179	0.85 (0.58–1.26)	0.435
	Secondary	0.68 (0.49–0.93)	0.016	0.88 (0.60–1.28)	0.496
	Higher	0.34 (0.13–0.88)	0.026	0.48 (1.77–1.35)	0.167

HHH, Household Head.

**Table 5:** Hierarchical regression analysis of the association of child characteristics with anemia among children aged 6 to 59 months in Zanzibar, Tanzania, based on the TDHS 2015.

Variables		Crude Odds Ratio (COR)		Adjusted Odds Ratio (AOR)	
		COR (95% CI)	*p*-Value	AOR (95% CI)	*p*-Value
**Child Characteristics**					
Age of child (months)	Q1	Ref		Ref	
	Q2	0.73 (0.43–1.24)	0.250	0.18 (0.12–0.28)	<0.001
	Q3	0.35 (0.21–0.58)	<0.001	0.30 (0.20–0.44)	<0.001
	Q4	0.19 (0.12–0.31)	<0.001	0.87 (0.66–1.13)	0.02
Gender of child	Male	Ref		Ref	
	Female	0.92 (0.72–1.17)	0.52	0.84 (0.64–1.09)	0.195
Birth weight (grams)	Above 2500 g	Ref		Ref	
	Below 2500 g	1.24 (0.69–2.21)	0.461	1.16 (0.62–2.17)	0.352
Place of delivery	Health facility	Ref		Ref	
	At home	1.06 (0.83–1.36)	0.622	0.95 (0.57–1.60)	0.863
Diarrhea	No	Ref		Ref	
	Yes	1.45 (0.97–2.16)	0.065	0.85 (0.55–1.33)	0.486
Symptoms of fever	No	Ref		Ref	
	Yes	1.24 (0.89–1.72)	0.188	1.13 (0.79–1.62)	0.485
Initiation of breastfeeding	Within an hour	Ref		Ref	
	After an hour	1.43 (1.03–1.99)	0.032	1.43 (0.98–2.05)	0.058
	After one day	0.72 (0.54–0.96)	0.025	1.0 (0.63–1.58)	0.988
MDD	No	Ref		Ref	
	Yes	1.0 (0.71–1.41)	0.985	0.61 (0.37–0.98)	0.045
MMF	No	Ref		Ref	
	Yes	2.66 (1.75–4.03)	<0.001	0.89 (0.49–1.63)	0.719
MAD	No	Ref		Ref	
	Yes	2.5 (1.14–5.36)	0.021	1.88 (0.68–5.20)	0.224
Deworming	No	Ref		Ref	
	Yes	0.74 (0.57–0.95)	0.020	1.14 (0.85–1.55)	0.377
Iron-rich food	No	Ref		Ref	
	Yes	1.55 (1.21–2.00)	0.001	1.18 (0.82–1.69)	0.375
Stunting	No	Ref		Ref	
	Yes	1.26 (0.95–1.67)	0.116	0.96 (0.67–1.36)	0.815
Wasting	No	Ref		Ref	
	Yes	1.56 (0.93–2.63)	0.094	0.91 (0.48–1.71)	0.768
Underweight	No	Ref		Ref	
	Yes	1.81 (1.24–2.63)	0.002	1.54 (0.94–2.52)	0.025

MDD, minimum dietary diversity; MMF, minimum meal frequency; MAD, minimum acceptable diet.

## Data Availability

The data for this study is available upon request from the TDHS portal (www.dhsprogram.com, access date: 16 January 2020).
